# Classic Galactosemia: Clinical and Computational Characterization of a Novel *GALT* Missense Variant (p.A303D) and a Literature Review

**DOI:** 10.3390/ijms242417388

**Published:** 2023-12-12

**Authors:** Giovanna Forte, Antonia Lucia Buonadonna, Antonino Pantaleo, Candida Fasano, Donatella Capodiferro, Valentina Grossi, Paola Sanese, Filomena Cariola, Katia De Marco, Martina Lepore Signorile, Andrea Manghisi, Anna Filomena Guglielmi, Simonetta Simonetti, Nicola Laforgia, Vittoria Disciglio, Cristiano Simone

**Affiliations:** 1 Medical Genetics, National Institute of Gastroenterology-IRCCS “Saverio de Bellis” Research Hospital, 70013 Castellana Grotte, Italy; forte.labsimone@gmail.com (G.F.); lucia.buonadonna@irccsdebellis.it (A.L.B.); pantaleo.labsimone@gmail.com (A.P.); fasano.labsimone@gmail.com (C.F.); grossi.labsimone@gmail.com (V.G.); sanese.labsimone@gmail.com (P.S.); filo.cariola@irccsdebellis.it (F.C.); demarco.labsimone@gmail.com (K.D.M.); leporesignorile.labsimone@gmail.com (M.L.S.); andrea.manghisi@irccsdebellis.it (A.M.); floranna.guglielmi@irccsdebellis.it (A.F.G.); 2 Section of Neonatology and Neonatal Intensive Care Unit, Department of Interdisciplinary Medicine, “Aldo Moro” University of Bari, 70121 Bari, Italy; dottcapodiferro@virgilio.it (D.C.); nicola.laforgia@uniba.it (N.L.); 3 Clinical Pathology and Neonatal Screening, Azienda Ospedaliera Universitaria Policlinico-Giovanni XXIII, 70124 Bari, Italy; simonetta.simonetti@policlinico.ba.it; 4 Medical Genetics, Department of Precision and Regenerative Medicine and Jonic Area (DiMePRe-J), University of Bari Aldo Moro, 70124 Bari, Italy

**Keywords:** GALT, classic galactosemia, novel missense variant

## Abstract

Classic galactosemia is an autosomal recessive inherited liver disorder of carbohydrate metabolism caused by deficient activity of galactose-1-phosphate uridylyltransferase (GALT). While a galactose-restricted diet is lifesaving, most patients still develop long-term complications. In this study, we report on a two-week-old female patient who is a compound heterozygote for a known pathogenic variant (p.K285N) and a novel missense variant (p.A303D) in the *GALT* gene. Segregation analysis showed that the patient inherited the p.K285N pathogenic variant from her father and the p.A303D variant from her mother. A bioinformatics analysis to predict the impact of the p.A303D missense variant on the structure and stability of the GALT protein revealed that it may be pathogenic. Based on this finding, we performed a literature review of all *GALT* missense variants identified in homozygous and compound heterozygous galactosemia patients carrying the p.K285N pathogenic variant to explore their molecular effects on the clinical phenotype of the disease. Our analysis revealed that these missense variants are responsible for a wide range of molecular defects. This study expands the clinical and mutational spectrum in classic galactosemia and reinforces the importance of understanding the molecular consequences of genetic variants to incorporate genetic analysis into clinical care.

## 1. Introduction

Galactosemia is a family of rare autosomal recessive hereditary liver disorders resulting from impairment of one of the four galactose metabolizing enzymes, i.e., galactokinase (GALK), galactose-1-phosphate uridylyltransferase (GALT), UDP-galactose 4-epimerase (GALE), and galactose mutarotase (GALM), that are involved in the Leloir pathway of galactose metabolism. The inability to metabolize galactose leads to hypergalactosemia [1]. Type 1 galactosemia (OMIM #230400) is caused by deficient activity of the GALT enzyme, which catalyzes the conversion of galactose-1-phosphate and UDP-glucose to UDP-galactose and glucose-1-phosphate (Glc-1-P). Type 1 galactosemia is the most frequent form of the disease with an overall prevalence of 1:16,000 to 1:50,000 live births in Western countries [2]. Based on GALT residual activity, galactosemia can be further classified into three clinical/biochemical phenotypes: (i) classic galactosemia (CG), which is characterized by the absence of GALT activity in erythrocytes and liver, (ii) clinical variant galactosemia, which is associated with a drastic reduction in GALT activity (1–10% residual activity in erythrocytes and/or liver), and (iii) biochemical variant galactosemia, which shows 15–33% residual GALT activity in erythrocytes [3]. The syndrome usually appears in the neonatal period after the ingestion of galactose from breast milk or infant formula. Clinical manifestations of CG may include jaundice, hepatomegaly, poor feeding, failure to thrive, hypoglycemia, Escherichia coli sepsis, and cataracts. Long-term complications such as cognitive impairment, ataxia, speech impairment, and ovarian dysfunction may also occur. The current standard of disease treatment is life-long dietary galactose restriction; however, this approach has proven inadequate in preventing long-term complications [4,5]. Therefore, novel and more effective treatment strategies are needed.

The *GALT* gene is located on chromosome 9p13 and comprises 11 exons [6]. To date, more than 350 *GALT* alterations have been reported, most of which are missense pathogenic variants [7,8]. Several disease-causing variants negatively impact GALT protein folding and stability [9]. In the European population, the most common missense variants are c.563A>G (p.Q188R) and c.855G>T (p.K285N), both of which have been associated with low GALT activity and poor prognosis [7]. Besides disease-causing variants, several *GALT* gene variants resulting in diverse levels of enzyme activity have been reported, including the Duarte variants (D1 and D2 alleles). In D1, also known as Los Angeles type, the c.940A>G (p.N314D) variant is in *cis* configuration (two genetic variants on the same allele) with the synonymous variant p.L218L (c.652C>T), inducing increased GALT activity due to overexpression of the enzyme. Instead, in D2, the c.940A>G variant occurs on the same allele with three intronic variants (c.378-27G>C, c.507+62G>A, and c.508-24G>A) and with the deletion of four bases in the promoter region (5′UTR-119_-116delGTCA), which cause decreased enzymatic activity [3,10].

In this study, we describe a two-week-old female patient who is a compound heterozygote for the known pathogenic variant c.855G>T (p.K285N) and a novel missense variant in the *GALT* gene. In an effort to evaluate the effects of this novel variant on protein function and stability, we performed an in silico analysis. Moreover, to gain further insight into the genotype–phenotype correlation of *GALT* pathogenic variants, we carried out a literature review of the clinical and molecular features of all missense variants reported as homozygous and compound heterozygous with c.855G>T (p.K285N) in galactosemia patients.

## 2. Results

### 2.1. Screening Procedures and Clinical History

A female neonate born by full-term normal vaginal delivery was admitted on day 7 of life with hypotonia, loss of appetite, and projectile vomiting that occurred after every feeding. There were no prenatal or perinatal problems (Apgar score 9–10). Upon admission to the hospital, hypoglycemia and jaundice were detected. Liver function tests showed abnormal values for total bilirubin (24 mg/dL, normal values 0.3–1.2 mg/dL), direct bilirubin (2.5 mg/dL, normal values < 0.3 mg/dL), aspartate aminotransferase (AST, 269 U/L, normal values < 34 U/L), and alanine aminotransferase (ALT, 136 U/L, normal values 10–49 U/L). Based on these results, galactosemia was suspected, and specific tests on dried blood samples were performed. Total galactose was 2680.37 µmol/L (normal values < 490 µmol/L), and a significant reduction in GALT activity (<2.5 U/dL, normal values > 6.7 U/dL) was detected in erythrocytes. Therefore, the patient was transitioned from milk to lactose-free enteral nutrition, and galactosemia tests were performed on her parents. Total galactose in the index case’s father and mother was low but still within the normal range (88.19 and 99.38 µmol/L, respectively). Normal total galactose values for adults range from 78 to 182 µmol/L according to our laboratory data obtained from individuals not harboring *GALT* germline pathogenic variants. Biochemical analysis of GALT activity in erythrocytes failed to show pathological enzyme activity values (11 U/dL in the mother and 8.3 U/dL in the father). However, these values were about 50% lower compared to our laboratory data obtained from adult individuals not harboring *GALT* germline pathogenic variants (16–20 U/dL). This is consistent with data from the literature, according to which GALT activity is reduced by approximately 50% in individuals heterozygous for *GALT* pathogenic variants [11,12].

### 2.2. Genetic Analysis

**Figure 1:**
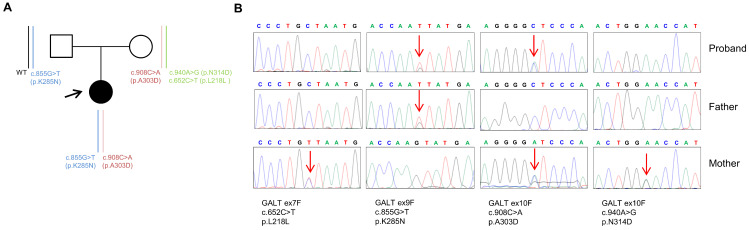
(**A**) Pedigree of the family involved in this study. Squares indicate men and circles indicate women. The arrow indicates the index case. Black-filled symbols denote individuals with galactosemia, and unfilled symbols indicate unaffected individuals. The vertical bars represent the *GALT* gene alleles segregating with the disease. (**B**) Sequencing electropherograms of genomic DNA from the index patient and her parents for all variants detected in the *GALT* gene.

Given the clinical data of the index case, her genomic DNA was extracted, and the *GALT* gene was analyzed by Sanger sequencing. This analysis identified two variants: c.855G>T (p.K285N) and c.908C>A (p.A303D) (Figure 1A,B).

The p.K285N variant was found to be rare in global population databases (gnomAD [13], 1000 Genomes Project [14], Exome Aggregation Consortium [15]) and has been reported in multiple individuals diagnosed with CG when found in *trans* configuration (two genetic variants on different alleles) with another pathogenic variant [16]. The p.A303D variant was also found to be rare since it was not listed in global population databases. Moreover, this variant has never been reported in major disease-associated databases (HGMD Professional [8], ClinVar [17], Mastermind [18]). Segregation analysis showed that the c.855G>T (p.K285N) variant was inherited from the father, while the c.908C>A (p.A303D) was inherited from the mother. Furthermore, full genetic testing of the *GALT* gene performed on the mother’s DNA revealed the presence of a c.652C>T nucleotide change (p.L218L) in *cis* with the c.940A>G (p.N314D) missense substitution (D1 Duarte variant), which were not identified in the index case’s DNA (Figure 1B). This analysis of the mother’s DNA excluded the presence of the three intronic variants (c.378-27G>C, c.507+62G>A, and c.508-24G>A) and the deletion of four bases in the promoter region (5′UTR-119_-116delGTCA) of the *GALT* gene that are associated with the D2 Duarte variant. The *GALT* variants identified in the index case were classified according to the American College of Medical Genetics and Genomics (ACMG) and the Association of Molecular Pathology (AMP) variant classification scheme [19]. Based on these criteria, the *GALT* (c.855G>T, p.K285N) variant was classified as pathogenic and the *GALT* (c.908C>A, p.A303D) variant was classified as likely pathogenic.

### 2.3. In Silico Analysis of the Structural Impact of the GALT A303D Missense Variant

**Figure 2:**
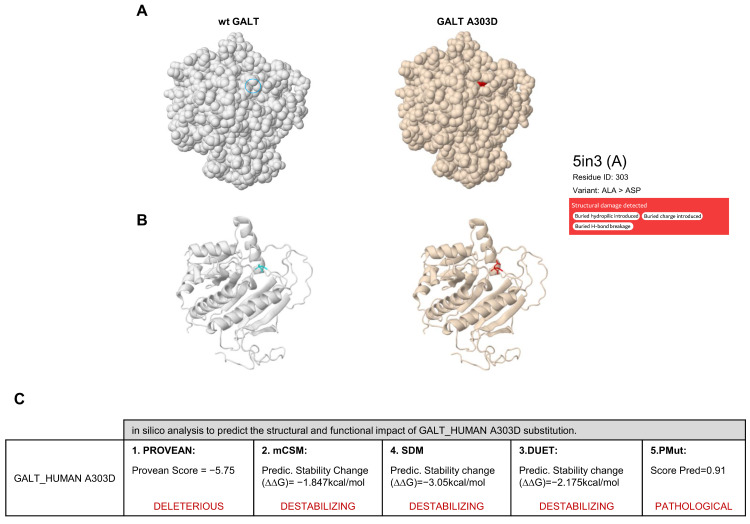
In silico analysis of GALT A303D variant. (**A**,**B**) Missense 3D analysis. Spacefill (**A**) and cartoon (**B**) structures of wild-type (wt) GALT (**left**) and GALT A303D variant (**right**). The GALT PDB entry considered for the analysis is 5in3. The wt residue Ala is indicated with a blue circle (**A**) or in blue (**B**), while the mutant residue Asp is indicated in red. (**C**) Results of the in silico prediction analysis of the structural and functional impact of the A303D substitution using the indicated computational tools.

Prediction analysis of the effects of the A303D missense substitution on the structure and function of the GALT protein was performed using the Missense3D database [20]. The Missense3D algorithm is based on a computational prediction method that uses protein structure information, either solely or in conjunction with sequence-based characteristics, to generate accurate estimates providing high true positive rates and low false positive rates. In particular, the tool is based on the modeling of protein tertiary 3D structures that are annotated in the PDB database and is capable of proteome-wide prediction of local protein properties, including regular secondary structures, residue burying, transmembrane-spanning sections, and disordered regions [20]. This prediction analysis suggested that the A303D substitution impacts GALT stability and may cause three different structural damaging effects: (i) it replaces a buried hydrophobic residue (ALA) with a hydrophilic residue (ASP); (ii) it replaces a buried uncharged residue (ALA) with a charged residue (ASP); and (iii) it disrupts all side-chain H-bond(s) and main-chain H-bonds formed by a buried ALA residue (Figure 2A,B). To corroborate the structural and functional impact of the A303D substitution suggested by Missense3D analysis, we used other computational tools based on different methodologies, such as PROVEAN (Protein Variation Effect Analyzer) [21], mCSM (mutation Cutoff Scanning Matrix) [22], SDM (Site-Directed Mutator) [23], DUET [24], and PMut (Prediction of Mutation) [25]. The scores resulting from this in silico meta-analysis confirmed the predicted destabilizing effect of the A303D missense substitution on GALT protein structure (Figure 2C).

### 2.4. Literature Review

**Figure 3:**
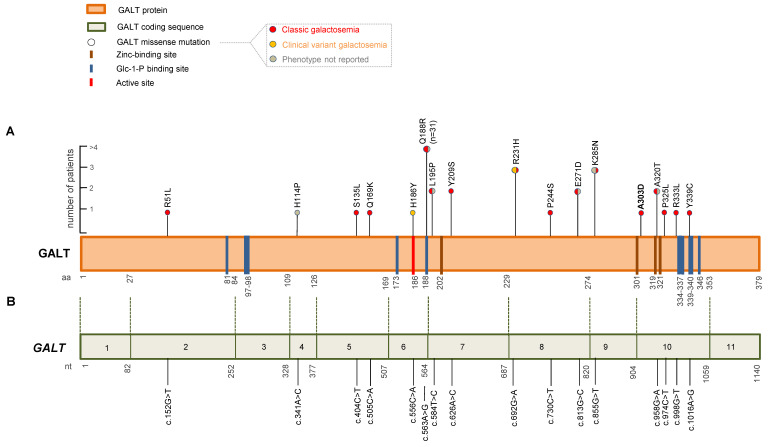
Graphical representation of GALT missense variants. (**A**) Lollipop graph of GALT protein variants. Distribution of *GALT* missense variants reported as homozygous and compound heterozygous with the c.855G>T (p.K285N) variant in galactosemic patients with clinical information identified in our literature meta-analysis and the present study. Vertical color bars in the amino acid sequence indicate functional sites: zinc-binding sites (brown), Glc-1-P (Glucose-1-phosphate) binding sites (blue), and active site (red). The galactosemia phenotypes associated with the missense variants are also reported: classic galactosemia (red), clinical variant galactosemia (orange), and phenotype not reported (gray). The scale bar on the left indicates the number of patients carrying each *GALT* missense variant. (**B**) Schematic representation of *GALT* coding sequence (NM_000155.4) and distribution of *GALT* missense variants.

To explore the association between amino acid alterations in the GALT protein and clinical manifestations of galactosemia, we performed a literature review of all *GALT* gene missense variants reported in homozygous and compound heterozygous patients with the c.855G>T (p.K285N) variant on Mastermind Professional. We only considered variants resulting in clinical manifestations. This analysis identified 54 patients with a total of 16 *GALT* missense variants (Figure 3A,B, Table S1). Of these, 3/54 (5.6%) were homozygous for c.855G>T (p.K285N), while 51/54 (94.4%) were compound heterozygous. Most of the patients (31/54, 57.4%) carried the c.563A>G (p.Q188R) variant, while the remaining 20 patients (37%) harbored 14 missense variants (Table S1). Specifically, the c.584T>C (p.L195P), c.626A>C (p.Y209S), c.813G>C (p.E271D), and c.958G>A (p.A320T) variants were found in two patients (3.7%) each, while the c.692G>A (p.R231H) variant was found in three patients (5.5%). The remaining 9 patients harbored 9 different *GALT* missense variants (p.R51L, p.H114P, p.S135L, p.Q169K, p.H186Y, p.P244S, p.P325L, p.R333L, and p.Y339C) (Figure 3A,B, Table S1). Overall, our analysis revealed that the missense variants were evenly distributed throughout the gene (Figure 3A,B). Galactosemia classification was reported for only 36 out of 54 patients. Most of the patients had a CG phenotype (33/36), while the remaining three patients developed clinical variant galactosemia. Furthermore, clinical data on liver function were reported for only 21/54 patients. Of these, 13/21 (61.9%) had hepatic damage.

## 3. Discussion

The spectrum of *GALT* pathogenic variants is highly heterogeneous. Most of them are missense variants causing changes in protein stability and folding, which result in a dysfunctional GALT enzyme [26,27]. Although early diagnosis and the current standard of care based on dietary interventions have led to an improvement in the quality of life of CG patients, new effective therapies are needed to prevent long-term debilitating complications. In this context, the study of disease-causative *GALT* genetic variations and their impact on protein function and stability is important to support the design of new therapeutic strategies [28].

In the current study, we report the clinical and molecular data of a novel *GALT* missense variant (c.908C>A, p.A303D) identified in a two-week-old female newborn with CG who is a compound heterozygote for the *GALT* pathogenic missense variant (c.855G>T, p.K285N). c.855G>T (p.K285N) is the second most frequent *GALT* genetic variant in European patients, accounting for 26–34% of galactosemia alleles. It is associated with partial loss (50%) of GALT activity in heterozygous patients and complete loss in homozygous patients [11,12]. Moreover, it has been shown to cause instability in protein structure [29].

Genetic analysis of the index case’s parents identified the c.855G>T (p.K285N) variant in the father and the c.908C>A (p.A303D) variant in the mother, both parents being heterozygous and asymptomatic carriers of these genetic alterations. Furthermore, in addition to the c.908C>A (p.A303D) variant, two other *GALT* gene variants (c.940A>G (p.N314D) and c.652C>T (p.L218L)) were identified in *cis* (D1 Duarte variant) in the mother’s DNA. Of note, D1 alleles carrying these variants have been reported to be associated with normal or above-normal GALT activity [30]. Consistently, GALT activity was higher in the mother of the index case than in the father, suggesting that the deleterious effect of the novel c.908C>A (p.A303D) variant may be compensated by the D1 allele.

To explore the effect of the c.908C>A (p.A303D) variant on GALT protein function and structure, we performed in silico prediction analyses.

A previous study on GALT crystal structure showed that this enzyme adopts a homodimeric configuration with two active sites formed by residues (H186) belonging to both subunits. Moreover, one zinc-binding site has been found per monomer, which is contributed by residues E202, H301, H319, and H321, while the binding site for Glc-1-P is constituted by residues N97, K334, F335, V337, Y339, E340, and Q346 on one chain and is complemented by residues N173 and Q188 on the other chain [29]. Additionally, N97 participates with Q188 in uridine monophosphate (UMP) and Glc-1-P binding [29]. As in all metalloenzymes, zinc-binding residues are critical for GALT enzymatic activity as they structurally stabilize the active site, and more generally they are important for the stabilization of the entire protein structure and its dimerization [29,31]. The protein residues that directly bind the zinc ion form a functional charge/dipole complex, whose polarization may modulate electrophilicity and consequently protein reactivity. Typically, these dipoles are surrounded by an outer shell of conserved amino acids that stabilize the zinc–protein complex by sustaining the electron deformation caused by charge/dipole polarization [32]. Intriguingly, A303 is located two amino acids downstream of the zinc-binding site H301; thus, it can be hypothesized that the substitution of an alanine (non-polar amino acid) for an aspartate (polar and charged amino acid) may impact the electrophilicity and functional polarization required for GALT biochemical reactivity. These pieces of evidence prompted us to analyze in silico the structural impact of the A303D missense substitution in the GALT protein using different in silico prediction tools, such as Missense3D [20], PROVEAN [21], mCSM [22], SDM [23], DUET [24], and PMut [25].

The results of this analysis consistently pointed to a direct impact of the A303D substitution on GALT structural stability and, therefore, on its enzymatic activity.

To further our understanding of the correlation between GALT amino acid alterations and clinical manifestations of galactosemia, we performed a literature review of compound heterozygous galactosemia patients carrying the c.855G>T (p.K285N) substitution in association with other pathogenic missense variants.

To date, nearly 360 pathogenic variants affecting the *GALT* gene have been described in galactosemia patients, the vast majority of which (69%) are missense variants, while 22.4% are loss-of-function variants (nonsense alterations, small deletions, small insertions, and gross rearrangements), and only 8.6% have been reported to cause or potentially cause impaired splicing (HGMD Professional) [8].

Our literature analysis revealed that 16 pathogenic *GALT* missense variants have been identified as homozygous and compound heterozygous with the c.855G>T (p.K285N) substitution in 54 patients. These 16 pathogenic *GALT* missense variants were distributed throughout the *GALT* sequence.

Interestingly, our analysis revealed that 34 out of 54 (63%) patients were compound heterozygotes for the c.855G>T (p.K285N) variant and another missense variant affecting residues (Q169, Q188, R333, and Y339) that are located close to the Glc-1-P binding sites. Among these patients, 22 out of 34 were described clinically and developed the classic form of the disease. Most of these amino acid changes are expected to cause a range of molecular defects, including altered substrate binding (p.Q188R), impaired dimerization (p.Q169K), and misfolding (p.Q188R, p.R333L) [29,33].

As regards the missense variant p.Y339C, which involves one of the Glc-1-P binding sites, no relevant data on its functional impact on disease phenotype have been reported in the literature.

In a previous study, the missense alteration p.H186Y was identified in addition to the p.K285N variant in a patient with the clinical variant form of galactosemia [34]. Notably, H186 is located in the GALT enzyme active site and participates in the covalent linkage with the UMP group of the uridine diphosphate-glucose (UPD-Glc) substrate, leading to the release of Glc-1-P [29]. The p.H186Y variant has been predicted to have a negative impact on substrate binding [29]. Considering the relevant functional role of the H186 residue, the mild disease presentation in the adult patient carrying the p.H186Y variant was unexpected.

Interestingly, our analysis revealed that 7 out of 54 (13%) galactosemia patients harbored the c.855G>T (p.K285N) pathogenic variant in addition to missense variants affecting residues (L195, Y209, A320, P325) located close to zinc-binding sites. Among these patients, 5 out of 7 were described clinically and had the classic form of the disease. Most of these amino acid changes are expected to cause molecular defects, including impaired dimerization (p.Y209S) and misfolding (p.L195P, p.P325L) [29]. For what concerns the p.A320T variant, which was identified in two galactosemia patients, although it is located between two zinc-binding site residues (H319 and H321), no data about its functional effect on disease pathogenesis have been reported to date in the literature.

All the other pathogenic variants identified in our literature analysis (p.R51L, p.H114P, p.S135L, p.R231H, p.P244S, p.E271D, and p.K285N) are located far from the zinc- and substrate-binding sites. They were identified in twelve galactosemia patients, six of which were described clinically and had the classic form of the disease [35,36,37]. Some of these missense variants (p.H114P, p.S135L, p.P244S, p.E271D, and p.K285N) were predicted to have a deleterious effect on protein folding [29], while the p.R51L variant was predicted to have a dramatic impact on intersubunit relationships, impairing correct dimerization and reducing GALT activity [33]. The p.R231H variant was found in three patients, one of which developed the classic form of the disease while the remaining two had the clinical variant form [34]. A previous study investigating the abundance and activity of GALT protein in galactosemia patients revealed that the p.R231H missense substitution is associated with a marked reduction in protein levels and close to null GALT activity [38]. Thus, further studies are needed to clarify the role of this variant in the milder clinical manifestations observed in affected patients.

Overall, our literature review revealed that the *GALT* K285N missense pathogenic variant, which was identified in compound heterozygosity with the novel variant A303D in our index case, has been found in association with several other *GALT* missense variants in patients with galactosemia. In particular, the studies considered in this analysis revealed that most of these missense variants, whether involving amino acids located close to functional protein sites or far from them, are associated with a range of molecular defects, including altered substrate binding (p.H186Y, p.Q188R), impaired dimerization (p.R51L, p.Q169K, p.Y209S), and misfolding (p.S135L, p.Q188R, p.L195P, p.R231H, p.P244S, p.E271D, p.K285N, p.P325L, p.R333L).

Most of the homozygous and compound heterozygous patients harboring the *GALT* K285N variant developed the classic form of the disease. In addition, our analysis showed that acute liver failure was found in 61.9% of these patients during the first weeks of life. Interestingly, our index case also presented with the severe form of galactosemia in association with acute liver failure. The novel p.A303D variant identified in our index case is predicted to negatively impact GALT protein structure and stability, like most of the missense variants affecting amino acid residues located close to zinc-binding sites. Taken together, these observations support the potential pathogenic relevance of the novel p.A303D variant identified in this study.

## 4. Materials and Methods

### 4.1. Screening Procedures

Biochemical evaluation of galactosemia was performed on the index case during her first days of life (day 7). Quantitative determination of galactose and galactose-1-phosphate concentrations and GALT enzymatic activity was carried out in blood specimens dried on filter paper using the GSP^®^ Neonatal GALT kit and the Neonatal GALT kit, respectively (both from Revvity, PerkinElmer, Inc., Waltham, MA, USA) according to the manufacturer’s instructions.

The study protocol was approved by our Institutional Ethics Committees (Comitato Etico Indipendente Azienda Ospedaliero-Universitaria “Consorziale Policlinico”, Bari, Italy). Written informed consent was obtained from a parent and/or legal guardian of the index case. All the procedures were performed in agreement with the guidelines of the Helsinki Declaration on Human Experimentation.

### 4.2. Genetic Analysis

Genomic DNA was extracted from peripheral blood with the QIAamp DNA Blood Mini Kit (Qiagen, Hilden, Germany) according to the manufacturer’s instructions. Genetic variant identification was conducted by Sanger sequencing. The coding regions of the *GALT* gene (NM_000155.4) including intron/exon boundaries were amplified as previously reported by Leslie et al. and Elsas et al. [6,39]. The promoter region of the GALT gene was analyzed as previously reported by Kozak et al. [40]. PCR sequencing and capillary electrophoresis were performed on an Applied Biosystems 3130 Genetic Analyzer (Thermo Fisher Scientific, Scientific, Waltham, MA, USA). Genetic variants were confirmed in independently amplified PCR products.

The global population frequency of the identified *GALT* gene variant was retrieved from the 1000 Genome [14] (http://www.internationalgenome.org/1000-genomes-browsers/; accessed on 1 September 2023), dbSNP [41] (https://www.ncbi.nlm.nih.gov/snp/; accessed on 1 September 2023), gnomAD [13] (https://gnomad.broadinstitute.org/; accessed on 1 September 2023), and NHLBI Exome Sequencing Project (ESP) [15] (https://evs.gs.washington.edu/EVS/; accessed on 1 September 2023) databases.

Moreover, the Mastermind [18] (https://www.genomenon.com/mastermind/; accessed on 1 September 2023), HGMD Professional [8] (https://digitalinsights.qiagen.com/products-overview/clinical-insights-portfolio/human-gene-mutation-database/; accessed on 1 September 2023), and ClinVar [17] (https://www.ncbi.nlm.nih.gov/clinvar/; accessed on 1 September 2023) databases were interrogated to assess the pathogenicity of the identified variant. The variant was classified according to the American College of Medical Genetics and Genomics (ACMG) and Association of Molecular Pathology (AMP) variant classification scheme [19].

### 4.3. In Silico Prediction Analysis

To investigate the functional impact of the c.908C>A (p.A303D) variant, we assessed its effect on the molecular structure of the human GALT (hGALT) protein (PDB entry: 5in3; UniProt entry: P07902, https://www.uniprot.org/uniprotkb/P07902/entry; accessed on 1 September 2023) by in silico prediction analysis using various computational tools based on different methodologies.

We first used the Missense3D database (http://missense3d.bc.ic.ac.uk:8080/; accessed on 1 September 2023), a valuable server for predicting the structural consequences of missense variations on protein structure [20]. The Missense3D tool uses a set of 16 structural features to predict the impact of amino acid substitutions. These features include disruption of a salt bridge, breakage of disulfide bonds, alteration of buried hydrogen bonds, changes in cavity properties, alterations in buried/exposed residues, introduction of buried glycine or proline, introduction or replacement of buried charges, switches in buried charges, introduction of buried hydrophilic residues, replacement of cysteine with proline, and alterations in secondary structure. Based on these features, Missense3D classifies substitutions as either “damaging” or “neutral” [20]. To confirm Missense 3D results, we performed an in silico meta-analysis using PROVEAN [21] (http://provean.jcvi.org/index.php; accessed on 10 November 2023), mCSM [22] (https://biosig.lab.uq.edu.au/mcsm; accessed on 10 November 2023), SDM [23] (https://biosig.lab.uq.edu.au/sdm; accessed on 10 November 2023), DUET [24] (https://biosig.lab.uq.edu.au/duet/; accessed on 10 November 2023), and PMut [25] (https://mmb.irbbarcelona.org/PMut/; accessed on 10 November 2023). PROVEAN is a software based on a computational approach for obtaining pairwise sequence alignment scores that allow the generation of precomputed predictions for 20 single amino acid substitutions and a single amino acid deletion at every amino acid position of all human and mouse protein sequences [21]. mCSM uses missense graph-based signatures that encode atom-to-atom distance patterns to represent the protein residue environment and train predictive models. Each missense alteration is thus represented as a signature vector, which is used to train and test predictive machine-learning methods for regression and classification tasks [22]. SDM is based on the statistical and potential energy variation (∆∆G), which calculates a stability score from environment-specific amino-acid substitution frequencies within homologous protein families. In our case, this corresponds to the free energy difference between wild-type GALT and the GALT A303D variant [23]. DUET is a web server that combines the results of two complementary approaches (mCSM and SDM) in an optimized predictor using Support Vector Machines (SVMs) to produce a consensus prediction. In comparison to either method alone, this method improves overall prediction accuracy and performs as well as or better than comparable methods [24]. PMut predicts the pathological character of single point amino acidic variations quickly and accurately (approximately 80% success rate in humans). The program also allows for the rapid detection of mutational hot spots, which can be identified using one of three methods: (1) alanine scanning, (2) massive mutation, and (3) genetically accessible mutations. When available, the PMut server provides a graphical interface for Protein Data Bank (PDB) structures as well as a database containing hot spot profiles for all non-redundant PDB structures [25].

### 4.4. Literature Review

The literature review was performed by using the Mastermind Genomic Search Engine Professional, a comprehensive piece of software enabling us to search the literature for gene variants [18]. We considered all the *GALT* gene missense variants reported as homozygous and compound heterozygous with c.855G>T (p.K285N), resulting in clinical manifestations of galactosemia. We reviewed all the articles retrieved from the above search and collected the clinical information (i.e., sex, age at diagnosis, age at last follow-up, GALT enzymatic activity, clinical subtype of galactosemia, hepatocellular damage) of affected patients. Patients without clinical information were excluded.

## 5. Conclusions

Importantly, we identified a novel *GALT* gene missense variant (p.A303D) with a potential structural damaging effect in a patient with the classic form of galactosemia. Moreover, our findings provide insights into the correlation between the structural consequences of missense variants at specific GALT sites and the clinical manifestations of the disease. Overall, this study highlights the importance of integrating data from multiple sources, including patients’ clinical and genetic data, clinical data related to published germline genetic alterations, and data on the structural impact of protein variants, to implement a multidisciplinary approach allowing an estimation of the clinical impact of new missense variants and the incorporation of genetic analysis into clinical care.

## Data Availability

Data are contained within the article and supplementary materials.

## Supplementary Materials

The supporting information can be downloaded at: https://www.mdpi.com/article/10.3390/ijms242417388/s1. References [34,35,36,37,42,43,44,45,46,47,48,49,50,51] are cited in the Supplementary Material.
