# Supplementary material for: Classic Galactosemia: Clinical and Computational Characterization of a Novel GALT Missense Variant (p.A303D) and a Literature Review
Source: Int J Mol Sci. 2023 Dec 12;24(24):17388. doi: 10.3390/ijms242417388 (PMC10744227; doi:10.3390/ijms242417388)
Supplement: Supplementary file 1 [file ijms-24-17388-s001.zip › ijms-2701430-supplementary.pdf]

Supplementary Table S1. Clinical summary of homozygous and compound heterozygous patients harboring the *GALT* c.855G>T (p.K285N) gene variant

ID	Sex	Age at diagnosis (days)	Age at last follow-up (years)	Allele 1	Allele 2	Galactosemia subtype	Hepato-cellular damage	Other clinical manifestations	Reference
1	F		16	R51L	K285N	Classic		Ovarian dysfunction, learning difficulties	Boutron et al[34]
2	M		22	H114P	K285N			Learning difficulties, lack of professional training	Boutron et al[34]
3	-	135	4	S135L	K285N	Classic	Yes	Hyperbilirubinemia, cataracts	Garcia et la[35]
4	M		4	Q169K	K285N	Classic		Neonatal exchange transfusion	Milánkovics et al[42]
5	M		35	H186Y	K285N	Clinical variant			Boutron et al[34]
6	-			Q188R	K285N	Classic		Poor intellectual outcome	Welsink-Karssies et al[43]
7	-			Q188R	K285N	Classic		Movement disorder	Welsink-Karssies et al[43]
8	-			Q188R	K285N	Classic		Movement disorder, poor intellectual outcome	Welsink-Karssies et al[43]
9	F	8	6.5	Q188R	K285N		No	Speech delay, coagulopathy, hyperbilirubinemia	Ramadža et al[37]
10	F	22	14	Q188R	K285N		Yes	Learning difficulties, coagulopathy, cataracts	Ramadža et al[37]
11	M	26	16.5	Q188R	K285N		Yes	Behavioral problems, learning difficulties, residual cataract	Ramadža et al[37]
12	M	150	22	Q188R	K285N		Yes	Cataract, coagulopathy, failure to thrive, hyperbilirubinemia, sepsis	Ramadža et al[37]
13	M		20	Q188R	K285N	Classic	Yes	Apraxia of speech, cataracts, sepsis	Viggiano et al[36]
14	M		3	Q188R	K285N	Classic	Yes	White matter anomalies	Viggiano et al[36]
15	M	11680 (32 years)	34	Q188R	K285N	Classic	No	Moderate cerebellar atrophy, global developmental delay, sensorineural hearing loss and myopia	Lucas-Del-Pozo et al[44]
16	M		8	Q188R	K285N	Classic	No	Hyperbilirubinemia	Yuzyuk et al[45]
17	M		12	Q188R	K285N	Classic	Yes	Speech delay, developmental delay, sepsis	Yuzyuk et al[45]
18	F			Q188R	K285N		No	Hyperbilirubinemia, cataract, sepsis	Schulpis et al[46]
19	M			Q188R	K285N		No	Hyperbilirubinemia	Schulpis et al[46]
20	-	30	0.17 (2 months)	Q188R	K285N	Classic	Yes	Hyperbilirubinemia, cataract	Garcia et al[35]
21	M	180	33	Q188R	K285N			Intellectual disability	Gubbels et al[47]

22	M	7	29	Q188R	K285N			Movement disorder	Gubbels et al[47]
23	F	15	36	Q188R	K285N			Ovarian dysfunction	Gubbels et al[48]
24	M		29	Q188R	K285N			Intellectual disability, anxiety, dysarthria	Waisbren et al[49]
25	M		33	Q188R	K285N			Depression, anxiety, tremor	Waisbren et al[49]
26	F		36	Q188R	K285N			Depression, anxiety	Waisbren et al[49]
27	F		21	Q188R	K285N	Classic		Speech problem, hypergonadotropic hypogonadism, movement disorder	Milánkovics et al[42]
28	M		21	Q188R	K285N	Classic		Speech problem, neonatal exchange transfusion, movement disorder	Milánkovics et al[42]
29	F		18	Q188R	K285N	Classic		Speech problem, movement disorder	Milánkovics et al[42]
30	F		16	Q188R	K285N	Classic		Speech problem, movement disorder, cataracts	Milánkovics et al[42]
31	M		14	Q188R	K285N	Classic		Speech problem, neonatal exchange transfusion	Milánkovics et al[42]
32	M		13	Q188R	K285N	Classic		Prenatal diet, speech problem, movement disorder	Milánkovics et al[42]
33	F		5	Q188R	K285N	Classic		Speech problem	Milánkovics et al[42]
34	F		5	Q188R	K285N	Classic		Speech problem, streak gonad, movement disorder	Milánkovics et al[42]
35	F		-	Q188R	K285N	Classic		Neonatal exchange transfusion	Milánkovics et al[42]
36	F		-	Q188R	K285N	Classic		Neonatal exchange transfusion	Milánkovics et al[42]
37	M		21	L195P	K285N				Gubbels et al[47]
38	F			L195P	K285N	Classic	Yes	Coagulopathy, failure to thrive, hypoglycemia, movement disorder	Asteggiano et al[50]
39	F		21	Y209S	K285N	Classic		Ovarian dysfunction	Boutron et al[34]
40	-			Y209S	K285N	Classic		Severe hypoglycemia in the neonatal period; mental development: retarded	Zekanowski et al[51]
41	M		37	R231H	K285N	Classic		Delayed milestones, learning difficulties, intellectual disability, movement disorder	Boutron et al[34]
42	F		17	R231H	K285N	Clinical variant			Boutron et al[34]
43	F		8	R231H	K285N	Clinical variant			Boutron et al[34]
44	F		24	P244S	K285N	Classic	Yes	Apraxia of speech, cataract, white matter anomalies, sepsis	Viggiano et al[36]
45	M	21	14	E271D	K285N		No	Behavioral problems, speech delay,	Ramadža et al[37]

								learning difficulties, hyperbilirubinemia, cataracts, sepsis	
46	M		6	E271D	K285N	Classic	Yes	Cataracts	Viggiano et al[36]
47	M	10	4.5	K285N	K285N		Yes	Hyperbilirubinemia	Ramadža et al[37]
48	F	8	13	K285N	K285N		No	Learning difficulties, severe osteopenia, cataract	Ramadža et al[37]
49	F		4	K285N	K285N	Classic	Yes		Viggiano et al[36]
50	F			A320T	K285N		No	Hyperbilirubinemia	Schulpis et al[46]
51	-			A320T	K285N	Classic			Zekanowski et al[51]
52	M		26	P325L	K285N	Classic		Learning difficulties, delayed milestones, low-skilled profession, movement disorder	Boutron et al[34]
53	M		39	R333L	K285N	Classic		Low-skilled profession, learning difficulties, speech delay	Boutron et al[34]
54	M		14	Y339C−N314D	K285N	Classic		Learning difficulties, speech delay	Boutron et al[34]
